# Comparing Tear Film Viscosity between Sjögren and Non-Sjögren Dry Eye Disease

**DOI:** 10.3390/life13071484

**Published:** 2023-06-30

**Authors:** Hung-Yin Lai, Alexander Chen, Po-Chiung Fang, Hun-Ju Yu, Ming-Tse Kuo

**Affiliations:** 1 Department of Ophthalmology, China Medical University Hospital, China Medical University, Taichung City 40402, Taiwan; d24758@mail.cmuh.org.tw; 2 Department of Ophthalmology, Antai Medical Care Cooperation Antai Tian-Sheng Memorial Hospital, Pingtung City 92842, Taiwan; a111085@mail.tsmh.org.tw; 3 Department of Ophthalmology, Kaohsiung Chang Gung Memorial Hospital and Chang Gung University College of Medicine, Kaohsiung City 83301, Taiwan; fangpc@cgmh.org.tw (P.-C.F.); angelayu@cgmh.org.tw (H.-J.Y.); 4 School of Medicine, Chang Gung University, Taoyuan City 33302, Taiwan

**Keywords:** tear film viscosity, reflective light particles, tear film homeostasis, dry eye disease, Sjögren syndrome

## Abstract

This study aimed to compare tear film viscosity (TFV) in Sjögren and non-Sjögren dry eye diseases (DEDs). This was a cross-sectional observational study. A total of 68 DED patients were enrolled, including 32 patients with Sjögren syndrome (SS) and 36 without SS. TFV was assessed by a tear film analyzer and determined by the momentary moving speed (MMS; MMS (t) = α × t^−β^, t = time (s)) with its power-law fitting-derived parameters (α and β). Among the four indices of TFV (MMS (0.1 s), MMS (2.0 s), α, and β), the SS-DED patients had significantly lower MMS (0.1 s) (*p* = 2.01 × 10^−5^), α (*p* = 0.0375), and β (*p* = 0.0076). The SS-DED group also had significantly higher OSDI, lower central and nasal tear meniscus height (TMH), and higher OSS. MMS (0.1 s) was significantly correlated with nasal TMH and OSS (ρ = 0.2520, *p* = 0.0381 in nasal TMH; ρ = −0.3487, *p* = 0.0036 in OSS). Index β was not correlated with any non-TFV tests. In conclusion, MMS (0.1 s), α, and β are promising TFV indices in distinguishing SS-DED from non-SS-DED patients early. Among these TFV indices, lower MMS is the best alternative clue for detecting SS-DED.

## 1. Introduction

The tear film covering the ocular surface is a thin layer of complex biochemical compositions. It protects and lubricates the ocular surface [1]. Dry eye disease (DED), a multifactorial disease, may lead to unstable tear film followed by ocular surface injuries and irritation. Many suspected DED patients who present with DED-like symptoms without apparently disrupted tear film were often diagnosed with neurogenic pain [2]. However, these patients may have been misdiagnosed if the evaluation of tear film homeostasis is based on the classical DED tests, which are not comprehensive enough [3]. Therefore, a novel analytical method emphasizing tear film homeostasis may provide valuable information to these patients [2].

Tear film viscosity (TFV) plays a critical role in eye blinking. The viscosity represents the fluid resistance during the tear film spreading over the ocular surface. When the eyes open, the tear film spreads upward along the ocular surface with the eyelid movement. Tear film also establishes a protective interface in front of the ocular surface, in which sufficient viscosity is vital for maintaining the stability of the tear film [4]. Several studies demonstrated that artificial tears with higher cohesive viscosity could improve dry eye symptoms and signs more than those without [5,6,7]. Previous studies also showed that different compositions of soft contact lens solution would result in different viscosity [8,9]. Apart from composition, viscosity is also related to osmolality and pH level. Dalton et al. found that the higher-viscosity solutions have higher osmolality [8]. Pena-Verdeal et al. revealed that viscosity would also affect the pH level in soft contact lens solution [9]. Like soft contact lens solution, tear components, including protein, lipid, and mucin, were thought to be responsible for altering the TFV [10,11]. Previous studies suggested that the interaction of tear proteins contributes to TFV [10] and proposed that secretory mucins, such as MUC5AC, would interact with polar lipids to increase TFV [11]. Since TFV plays an essential role in reflecting the homeostasis of the tear film, TFV was adopted as a tear film marker for distinguishing DED patients from healthy control subjects [12,13].

The tear exhibited higher extensional viscosities at lower shear rates and higher spreading speeds [13]. Varikooty et al. pointed out that tear film spreading could indirectly reflect TFV [14]. Accordingly, the spreading speed of the tear film on the ocular surface can estimate the TFV, in which slower tear film spreading indicates higher friction or external viscosity between tears and the ocular surface [15,16]. Previously, we had established a method to quantify tear film spreading based on a popularly commercialized tear film analyzer, Keratograph^®^ 5M (K5M; Oculus, GmbH, Wetzlar, Germany) [16]. Briefly, we standardized the positional coordinate of each tear film particle on each K5M video-decomposed image frame with a time interval of 0.1 s. Reflective light particles on the cornea starting from the opening cycle of the blink for 1 s were tracked. The momentary moving speed (MMS) of a tear film particle at a specific moment was defined as the positional change from a frame to the next frame with a time interval of 0.1 s, and the power-law fitting for MMS data was used to extract two estimators (α and β) by MMS (t) = α × t^−β^, where α is the scaling factor, β is the power factor, and t is the time [16]. The parameters MMS, α, and β were used for TFV analysis. We concluded that this examination could help assess tear film homeostasis but had not yet confirmed the efficacy in distinguishing DED caused by different etiologies.

Sjögren syndrome (SS) is an autoimmune disorder characterized by exocrine gland dysfunction involving the lacrimal and salivary glands. Meibomian glands, a kind of sebaceous glands presenting in eyelids, could also be damaged by excessive lymphocyte infiltration, causing meibomian gland dysfunction. SS patients may exhibit more severe meibomian gland destruction than non-SS patients [17]. The meibomian glands secrete lipids that form the lipid layer of the tear film, which helps retard evaporation. When the meibomian gland is dysfunctional due to inflammatory infiltration, it can lead to disruption of tear film stability, resulting in DED. Compared to non-SS-DED, SS-DED patients risk serious eye complications, including ulcerative keratitis, scleritis, uveitis, and so on [18]. Thus, the ability to predict SS in DED patients by merely evaluating the tear film would be of tremendous value. Previous researchers found that patients with SS-DED had lower tear meniscus height (TMH), shorter tear break-up time, more severe ocular surface staining, and a larger area of meibomian gland atrophy than those without [19,20]. However, no single ocular index could differentiate SS from non-SS-DEDs.

The evaluation of TFV based on tear film spreading on the cornea is a noninvasive and emerging method for the dynamic assessment of tear film homeostasis [16]. We hypothesized that the TFV of SS-DED is higher than that of non-SS-DED. Therefore, this study aimed to identify the differences in the K5M-based TFV indices between SS and non-SS-DED patients and the potential indexes for early detecting SS from DED patients.

## 2. Materials and Methods

### 2.1. Participants

This investigation was a cross-sectional study in which Asian subjects were enrolled from October 2019 to March 2022 at Kaohsiung Chang Gung Memorial Hospital (CGMH). This study (Registration No. 201900954B0) was approved by the Institutional Review Board of CGMH and adhered to the tenets of the Declaration of Helsinki. This study included stably controlled dry eye subjects with ocular surface disease index (OSDI) > 13, Oxford staining score (OSS) > 1, or first noninvasive keratograph break-up time (NIKBUT first) < 10 s for at least once at enrollment [3]. These subjects were stably controlled with lubricants, and most SS-DED subjects were also treated with oral hydroxychloroquine. All enrolled DED subjects had tolerable or no ocular symptoms under the current treatment regimen, no filamentary keratitis, and no increased ocular punctate erosions over 3 months. DED subjects under 20 years of age, glaucoma, acute ocular inflammation, intraocular or eyelid surgery within six months, diabetes mellitus, pregnancy, or failure to complete all examinations were excluded. All participants were informed about the purpose and procedure of this study and signed the informed consent. All SS-DED subjects were diagnosed and confirmed by rheumatologists according to respective classification criteria [21,22,23] in CGMH. All non-SS-DED subjects were negative for anti-SSA/Ro and anti-SSB/La antibodies in serum. Thirty-two DED patients with SS and thirty-six without SS were enrolled for analysis.

### 2.2. Assessment Protocol

The right eye was selected for measurement when both eyes met the inclusion criteria, while the left eye was measured if the right eye did not meet the inclusion criteria. The masked technicians interpreted the examination procedure for the subject before each tear film test. After completing a dry eye questionnaire with Ocular Surface Disease Index (OSDI) [24], each subject received a series of ocular surface examinations with the K5M, including TMH [25,26,27,28,29], TFV [15,16,30,31], ocular surface redness [26,28,29,32], NIKBUT [25,26,27,28,29], and meibography [27,28,33]. Finally, each subject received cobalt blue light illuminated corneal photography after fluorescence dye staining to determine the corneal epitheliopathy [28,29,34]. The procedure and sequence of assessments were the same for each patient with the following order: OSDI, TMH, TFV, ocular surface redness, NIKBUT, meibography, and Oxford staining score. The technicians performed the questionnaire investigation and performed the ocular surface examinations with the K5M. The meibograde and Oxford staining scores of each subject were performed by the same doctor (Ming-Tse Kuo) at the outpatient clinic. All tear film viscosity via reflective light particles spreading on the cornea was analyzed by the same doctor (Hung-Yin Lai).

### 2.3. Evaluating the Subjective Severity of Dry Eye via the OSDI Questionnaire

The OSDI questionnaire consists of a total of twelve questions [24]. The score of each question ranges from 0 to 4 (none of the time to at all times) according to the frequency. By the number of ratings, the overall score is determined for all questions answered and separated by the total number of questions answered. The OSDI is on a scale of 0 to 100, with a higher score indicating more significant impairment. After a technician’s guidance, each patient completed the questionnaire independently. The score was measured and reported for each subject after completing the questionnaire.

### 2.4. Determination of Tear Volume on the Ocular Surface

TMH was evaluated under an infrared 880 nm light source to capture the lower tear film meniscus images. The measurement was performed for one second after each blink for each subject. Central TMH images captured by the K5M were measured perpendicular to the lid margin at the central point relative to the pupil center using an integrated ruler. Nasal and temporal TMHs were measured perpendicular to the lid margin at the nasal and temporal pupil limbus sites [25].

### 2.5. Assessment of Tear Film Viscosity via Reflective Light Particles Spreading on the Cornea

The TFV assessment was based on a K5M model, which adopts a two-diode white light source to illuminate the ocular surface and capture the video of flowing tear film particles on the cornea [16]. In brief, the first qualified blink cycle video was adopted for the assessment, and its decomposed image frames were used to analyze the trajectories of particles with a time interval of 0.1 s. By tracking three accessible reflective light particles on the cornea starting from the opening cycle of the blink for 1 s, the moving speeds of three reflective light particles were averaged to obtain each subject’s momentary moving speed (MMS). A power-law fit operation MMS (t) = α × t^−β^ was used to estimate the moving velocity of reflective light particles at every moment [16]. The early tear film spreading phase was defined as the time within 1 s, and the late tear film spreading phase was defined as the period equal to and after 1 s. The initial MMS was defined at t = 0.1 s (MMS (0.1 s)), and the final MMS was specified at t = 2 s (MMS (2 s)). We adopted four MMSs at the early phase (MMS (0.01 s), MMS (0.05 s), MMS (0.1 s), and MMS (0.5 s)), two MMSs at the late phase (MMS (1 s) and MMS (2 s)), α, and β as the indices for the TFV analysis. All of the TFV analysis was performed by the same doctor.

### 2.6. Determination of Tear Volume on the Ocular Surface

The ocular surface redness was evaluated by the K5M, which used the ring white light source to capture the ocular surface image under the primary gaze. After blinking, each patient was instructed to gaze straight ahead and focus on the fixation mark inside the camera. The six parameters of this examination were automatically calculated from the built-in software, including mean redness score, temporal bulbar scores, nasal bulbar scores, temporal limbal scores, nasal limbal scores, and the accessible area. These scores were estimated by the area percentage ratio between blood vessels and the rest of the scanned bulbar conjunctiva using a clinical grading scale of 0.0–4.0 in 0.1 steps [32].

### 2.7. Evaluation of Noninvasive Keratograph Break-Up Time (NIKBUT)

NIKBUT measured the time between a blink and the disruption of the rings reflected on the tear surface, which the device automatically detects. The K5M can display the result of the first noninvasive keratograph break-up time (NIKBUT first), the average noninvasive keratograph break-up time (NIKBUT avg), and the period of NIKBUT test (total assessable time) [25]. Patients were instructed to fixate centrally and forcefully suppress their blink as long as possible during a masked technician’s examination.

### 2.8. Evaluation of Meibomian Gland Dropout

The meibography was used to examine the upper and lower eyelid meibomian glands with a near-infrared illumination (840 nm diode light source) via K5M. The severity of the meibomian gland dropout was classified from degree 0 to 4 according to the meibograde proposed by Pult et al., with each degree of rise corresponding to a 25% loss of area in the meibomian gland [33]. A higher meibograde was selected for analysis if scores were obtained from the upper and lower eyelid meibography. The grading was performed by the same doctor at the clinic.

### 2.9. Evaluation of Oxford Staining Score

Oxford staining score was graded according to the Oxford Scheme [34], in which fluorescein stain was applied on the ocular surface, and then corneal staining was evaluated under absorption filters. Grade 0 (none) to 5 (all) was determined by the staining appearance, upon which confluent patches, pupillary area staining, and filaments would add another grade. The same doctor performed the grading at the clinic.

### 2.10. Sample Size Estimation

The sample size was estimated by an online sample size calculator [35]. Because no previous study has focused on comparing TFV between SS- and non-SS-DED, we calculated the sample size according to the difference in Oxford staining score between SS and non-SS-DED [36]. We adopted the significance level as 0.05, the desired power as 0.9, the standard deviation as 0.6, and the effect size as 0.5. Accordingly, each group had an estimated sample size of at least 24 eyes. Furthermore, the differences required in clinical studies for signs and symptoms of DED as well as the resulting sample sizes, have been proposed in the TFOS DEWS II Diagnostic Methodology report [3]. The minimum sample size per group is 15 in osmolarity, 3 in NIKBUT obtained from K5M, and 6–16 in phenol red thread test. In summary, a minimum of 24 participants per group is sufficient to obtain a statistical difference during evaluation.

### 2.11. Statistical Analysis

The MMS was estimated by Microsoft Excel 2016, where the add-in program, Solver, was used to perform the power-law fitting and extract the model parameters α and β. The Mann–Whitney U test measured the statistical differences in TFV parameters between the SS and non-SS-DED patients. The Spearman correlation coefficient determined the relationship between novel TFV parameters and commonly used parameters for DED. GraphPad Prism version 9.4.1 for Windows (GraphPad Software, San Diego, CA, USA) was used for the above statistical analysis. All values were presented as mean ± standard deviation (SD). *p*-value < 0.05 was considered statistically significant.

## 3. Results

### 3.1. Clinical Profile of Dry Eye Patients with and without Sjögren Syndrome

There was no significant difference in age (*p* = 0.089) (Table 1). Both SS- and non-SS-DED groups were predominantly female, but the sexual difference reached a statistical difference between these two groups (*p* = 0.0025). SS-DED subjects had significantly higher OSDI scores than those non-SS-DED subjects (*p* = 0.0033). Among these classical DED tests, SS-DED patients had significantly lower central TMH (*p* = 0.0065), lower nasal TMH (*p* = 0.0124), and higher OSS (*p* = 0.0455) than those of the non-SS-DED patients (Table 1).

### 3.2. Comparison of the Power-Law Fitting Curve between SS- and Non-SS-DED Subjects

**Figure 1:**
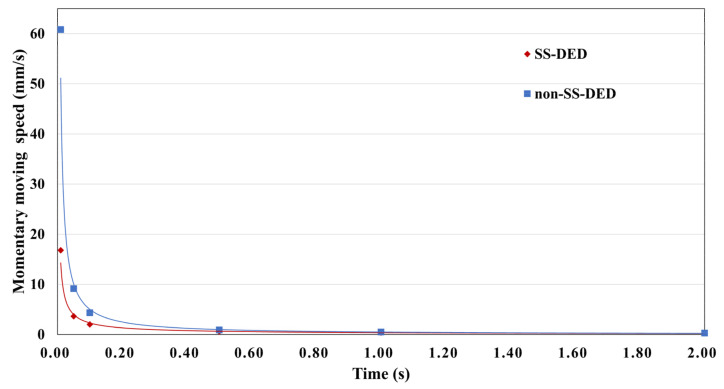
The trend of the averaged momentary moving speed changing with time in SS- and non-SS-DED patients. SS-DED = dry eye disease with Sjögren syndrome. non-SS-DED = dry eye disease without Sjögren syndrome.

**Figure 2:**
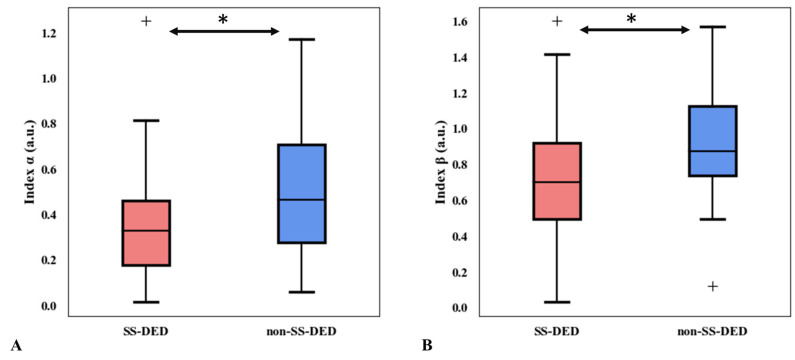
Comparison of the coefficient α and exponent β in the function of momentary moving speed between SS- and non-SS-DED subjects. (**A**) Index α (*p* = 0.0375). (**B**) Index β (*p* = 0.0076). SS-DED = dry eye disease with Sjögren syndrome. non-SS-DED = dry eye disease without Sjögren syndrome. a.u. = arbitrary unit. Each box was constructed of five parameters, including the median (Q2), lower, and upper quartiles (Q1, Q3), and lowest and highest data (Q1 − 1.5 × (Q3 − Q1), Q3 + 1.5 × (Q3 − Q1)). Outliers outside of the lowest and highest data were marked as +. Mann–Whitney U test was used for the statistical analysis. * *p* < 0.05.

A power-law fitting operation MMS (t) = α × t^−β^ was used to extract TFV markers based on the particle-tracking model for each subject. The profile of average MMS in SS- and non-SS-DED patients is shown in (Figure 1). The regression lines of the MMS approached a similar level as time progressed. Coefficient α, a scaling operator of the MMS function, was significantly lower (*p* = 0.0375) in SS-DED patients. Moreover, exponent β, a nonlinear trend-changing operator in the MMS function, was significantly lower (*p* = 0.0076) in SS-DED patients (Figure 2).

### 3.3. The Momentary Moving Speed of Reflective Light Particles at the Early and Late Tear Film Spreading Phases

The MMS of the SS-DED group was lower than that of the non-SS-DED group in the early tear film spreading phase, which implied that the SS-DED had greater TFV. Among the representative time points (0.01 s, 0.05 s, 0.1 s, 0.5 s, and 1.0 s), all MMSs of SS-DED patients were significantly lower than those of the non-SS-DED patients (Table 2), and index MMS (0.1 s) had the most remarkable statistical difference (*p* < 0.0001). Nevertheless, the MMS of the two groups were not significantly different at the 2 s time point in the late tear film spreading phase (Table 2).

### 3.4. Correlation between TFV Indices and Classical DED Parameters

**Figure 3:**
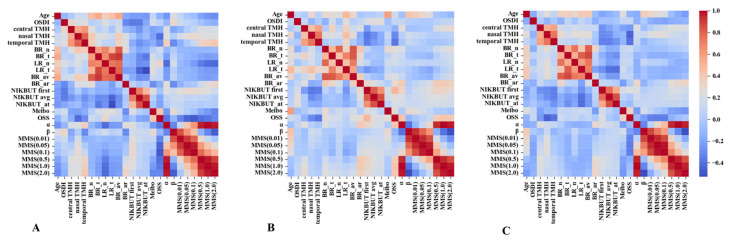
Spearman correlation matrix between the result of classical DED tests and the index of tear film viscosity in SS-DED, non-SS-DED, and all DED patients. (**A**) SS-DED patients. (**B**) Non-SS-DED patients. (**C**) All DED patients. DED = dry eye disease. SS-DED = dry eye disease with Sjögren syndrome. Non-SS-DED = dry eye disease without Sjögren syndrome. OSDI = ocular surface disease index. TMH = tear meniscus height. BR_*n* = nasal bulbar redness score. BR_t = temporal bulbar redness score. LR_n = nasal limbal redness score. LR_t = temporal limbal redness score. BR_av = mean redness score. BR_ar = assessable area of ocular redness test. NIKBUT first = first noninvasive keratograph break-up time. NIKBUT avg = average noninvasive keratograph break-up time. NIKBUT_at = assessable time of noninvasive keratographic break-up test. Meibo = meibograde. OSS = Oxford staining score. MMS = momentary moving speed. Red = a positive correlation; blue = a negative correlation.

Among SS-DED, non-SS-DED, and all DED patients, the pseudocolor patterns were similar but not identical (Figure 3). The index α was positively correlated with the MMS after 0.5 s. The index β was positively associated with the MMS before 0.5 s. For SS-DED patients (Figure 3A), the index α was positively correlated with the temporal TMH (ρ = 0.3850, *p* = 0.0296), while the index β was not associated with any standard parameter of DED. The MMS (0.1 s) was negatively correlated with the OSS with a statistical difference (ρ = −0.4357, *p* = 0.0126). For non-SS-DED patients (Figure 3B), the index α was positively correlated with the OSS (ρ = 0.3576, *p* = 0.0323), but indices β and MMS (0.1 s) were not associated with all standard DED parameters. For all DED patients (Figure 3C), the indices α and β were not correlated with any common DED parameter (Table 1), whereas MMS (0.1 s) was positively correlated with the nasal TMH (ρ = 0.2520, *p* = 0.0381) but was negatively correlated with the OSS (ρ = −0.3487, *p* = 0.0036).

## 4. Discussion

SS-DED patients generally present with a more unstable tear film and a poorer ocular surface when compared to non-SS-DED patients, even under stable control with long-term topical medications [36]. Ophthalmologists frequently diagnose SS patients earlier than rheumatologists because of patients’ dry eye presentations. Loss of tear film homeostasis is an essential feature in diagnosing DED [2]. However, determining tear film homeostasis requires a comprehensive assessment of the tear film and ocular surface [3]. Therefore, developing a new tear film test and confirming its utility in the early detection of SS from DED patients is crucial. This study is the first investigation to compare the TFV between SS- and non-SS-DED patients. We adopted a clinically available platform to estimate TFV [16], an indicator of tear film homeostasis. We demonstrated that indices of early MMS, α, and β of the TFV test significantly differed between SS- and non-SS-DED patients. SS-DED patients had significantly slower early MMS and higher TFV than non-SS-DED patients. Moreover, SS-DED patients had significantly lower values of indices α and β, in which index α was correlated with late MMS while index β was associated with early MMS. The index β had no association with standard DED parameters, making it a distinctive index in determining tear film homeostasis.

TFV plays an essential role in tear film homeostasis. Pandit et al. in vitro measured tear viscosity by a rotating rheometer in 1999, in which a large tear fluid volume was needed [37]. Parkin et al. resolved the large sample volume requirement and measured tear viscosity with a microviscometer, but the trapping laser of this equipment might locally heat the sample and absorb tears [38]. McDonnell et al. used a filament-stretching rheometry technique to obtain effective tear extensional viscosity [39]. However, the methods mentioned earlier all required the collection of tear fluids and in vitro analysis using sophisticated instruments to detect the internal or cohesive TFV, representing the intermolecular viscosity within the tears. Higher internal viscosity demonstrates faster tear film spreading on the ocular surface. Our method estimated the external TFV by determining the viscosity between the tear fluid and the ocular surface [16]. Higher external viscosity implied slower reflective light particles moving on the cornea, in which more friction between spreading tears and the cornea. More importantly, external TFV examinations can be implemented clinically and noninvasively with the commercialized K5M tear film analyzer, a repeatable instrument for dry eye evaluations [25,40].

The tear film mucin could be divided into secreted mucin and membrane-associated mucin. The secreted mucin ensures tears’ shear-thinning property and facilitates tears’ spreading, while the membrane-associated mucin keeps the ocular surface wettable [11]. Due to secreted mucin being related to tears’ shear-thinning properties and spreading, it might relate to TFV [11]. The most abundant secreted mucin, MUC5AC, had been used to differentiate SS- and non-SS-DED patients. Akpek EK et al. evaluated tear film biomarkers among SS- and non-SS-DED patients and found that tear MUC5AC was significantly lower in SS-DED patients [19]. In our study, SS-DED patients had significantly lower early MMS. Thus, the lower tear mucin level may lead to slower MMS in the early spreading phase. Consequently, the early MMS, acting as external TFV, could be a potential tear film hemostatic marker for the noninvasive assessment of tear film mucin level.

The SS-DED patients in this study had significantly higher OSDI than those of the non-SS-DED patients, which was compatible with the results of a previous study [41]. However, some researchers disagree with our results [19,42]. The subjective nature of the OSDI questionnaire, which is easily confounded by treatment, could be the reason.

The OSS was significantly higher in SS-DED patients in our study. Some prior studies showed that SS-DED patients had significantly more intense corneal and conjunctival staining than those non-SS-DED patients [19,41]. Their results were compatible with our findings, indicating more ocular surface damage in SS-DED patients. Hence, early detection of SS-DED patients for further treatment is important.

SS-DED patients had significantly lower central TMH, which was compatible with previous studies [43,44]. We further evaluated nasal and temporal TMHs. SS-DED patients had lower nasal and temporal TMH than non-SS-DED patients, but only the nasal TMH reached a statistical difference. A former study found that the conjunctival folds may influence the TMH, of which temporal conjunctival folds had a more significant correlation with the TMH than those of the nasal side [45]. Therefore, we propose using the central TMH as the standard measurement to avoid the influence of conjunctival fold. However, a future study may be needed to confirm our findings.

There was no significant difference in NIKBUT first between SS-DED and non-SS-DED patients in our study. However, earlier studies showed that SS-DED patients had shorter NIKBUT first compared to non-SS-DED patients [20,46]. There are two possible reasons for this discrepancy. First, our inclusion criteria enrolled many non-SS-DED patients with high tear film instability. Second, the accuracy of the NIKBUT test may be influenced by dry eye severity. Some researchers suggested that the NIKBUT test was more reliable and repeatable in the DED group than in the healthy group [25,47]. DED patients had reduced corneal sensitivity, resulting in less reflex tearing, minimizing the influence of examination [48]. Different presentations of tear film homeostasis between our SS-DED and non-SS-DED patients may interfere with the results of the NIKBUT test. Hence, a more comprehensive examination is needed for the dry eye evaluation.

Exploring the relationship between TFV indices and the parameters of standard DED tests, lower MMS (0.1 s) was significantly correlated with higher OSS in all DED patients (ρ = −0.3487, *p* = 0.0036), including SS-DED and non-SS-DED. This result implied that higher external TFV or lower internal TFV would compromise tear film stability and may result in more ocular surface damage. Therefore, TFV is an innovative tear film homeostatic marker that may provide an alternative clue about tear film stability.

There were a few limitations in this study. Firstly, there was a significant difference in gender between SS-DED and non-SS-DED groups. Both SS-DED and non-SS-DED groups were predominantly female, with a statistical difference. This sampling bias might be due to non-random sampling, and only a single medical center was included. The results were consistent with the previous finding that SS-DED patients were predominantly female [49]. To confirm the consistent performance of TFV between men and women in the non-SS-DED group, we further compared the TFV indices between 9 males and 27 females in the non-SS-DED group. There was no significant difference in age between these two groups. We found no gender difference in all TFV indices, including α, β, and MMS, in the early and late spreading phases (see Supplementary File S1). Hence, we believe that gender did not affect the TFV assessment. Secondly, several technicians performed the K5M examinations. However, all technicians were well-experienced and strictly followed the protocol to examine these DED patients. Moreover, a previous study reported suitable repeatability and reproducibility for a technician to obtain the results of TMH and NIKBUTs by K5M [25,40]. Thirdly, only the Asian population was enrolled. Ethnicity has been evaluated as a factor in dry eye disease, in which the Asian population appears to have a higher risk [50]. Future multicenter and prospective studies enrolling larger populations are required to verify the results of this study. Fourthly, only one eye was measured and analyzed, which was not a controlled sequence, as it was influenced by its availability. However, most studies about DED chose single eye for investigation and drew conclusions based on monocular results because of the high binocular correlation in tear film parameters [28,51,52].

## 5. Conclusions

In conclusion, the external TFV assessment is a promising tool for evaluating tear film homeostasis. The representative TFV indices, MMS (0.1 s), α, and β were significantly lower in SS-DED patients, providing an alternative hint to differentiate SS from DED patients. Among these TFV indices, lower MMS (0.1 s) is the best biomarker for helping physicians to detect SS-DED.

## Figures and Tables

**Table 1:** Demographic data of subjects.

Characteristics of Subjects	Dry Eye Disease with Sjögren Syndrome (*n* = 32)	Dry Eye Disease without Sjögren Syndrome (*n* = 36)	*p*-Value
Age (years)	61.38 ± 9.91	65.25 ± 6.80	0.089
Male:Female	0:32	9:27	0.0025 **
OSDI *^a^*	55.11 ± 24.01	38.56 ± 19.30	0.0033 **
TMH *^b^*			
Central	0.20 ± 0.22	0.21 ± 0.14	0.0065 **
Nasal	0.30 ± 0.18	0.42 ± 0.23	0.0124 *
Temporal	0.30 ± 0.18	0.36 ± 0.23	0.4777
Bulbar redness (a.u. *^c^*)			
Nasal bulbar score	1.53 ± 0.80	1.53 ± 0.61	0.7039
Temporal bulbar score	1.60 ± 0.69	1.51 ± 0.42	0.7414
Nasal limbal score	1.01 ± 0.70	1.09 ± 0.51	0.1118
Temporal limbal score	1.14 ± 0.60	1.10 ± 0.41	0.7718
Mean redness score	1.56 ± 0.67	1.50 ± 0.42	0.8259
Mean analyzed area	7.39 ± 2.80	8.26 ± 2.94	0.2113
Tear break-up time (s)			
NIKBUT first *^d^*	5.47 ± 3.10	4.79 ± 1.95	0.4654
NIKBUT avg *^e^*	7.73 ± 4.00	7.98 ± 4.52	0.7949
Assessable time	10.84 ± 5.04	12.05 ± 5.71	0.3789
Meibograde	2.34 ± 0.97	2.03 ± 0.77	0.1770
OSS *^f^*	1.22 ± 1.26	0.58 ± 0.77	0.0455 *

*^a^* OSDI, ocular surface disease index; *^b^* TMH, tear meniscus height; *^c^* a.u., arbitrary unit; *^d^* NIKBUT first, first noninvasive keratograph break-up time; *^e^* NIKBUT avg, average noninvasive keratograph break-up time; OSS *^f^*, Oxford staining score. * *p* < 0.05 and ** *p* < 0.01.

**Table 2:** Comparison of the momentary moving speed between SS- and non-SS-DED at representative time points.

Characteristics of Subjects	Dry Eye Disease with Sjögren Syndrome	Dry Eye Disease without Sjögren Syndrome	*p*-Value
MMS (0.01 s)	16.81 ± 18.82	60.82 ± 70.91	0.0002 **
MMS (0.05 s)	3.64 ± 2.90	9.16 ± 6.69	<10^−5^ ***
MMS (0.1 s)	2.01 ± 1.39	4.34 ± 2.49	<10^−5^ ***
MMS (0.5 s)	0.59 ± 0.37	0.91 ± 0.40	0.0028 **
MMS (1.0 s)	0.37 ± 0.27	0.50 ± 0.27	0.0375 *
MMS (2.0 s)	0.25 ± 0.23	0.29 ± 0.21	0.2983

MMS = momentary moving speed. Mann–Whitney U test was used for the statistical analysis. * *p* < 0.05, ** *p* < 0.01, *** *p* < 0.001.

## Data Availability

The data presented in this study are available upon request from the corresponding author.

## Supplementary Materials

The following supporting information can be downloaded at: https://www.mdpi.com/article/10.3390/life13071484/s1, File S1: Comparison of the momentary moving speed between male and female at representative time points.
