# Supplementary material for: Comparing Tear Film Viscosity between Sjögren and Non-Sjögren Dry Eye Disease
Source: Life (Basel). 2023 Jun 30;13(7):1484. doi: 10.3390/life13071484 (PMC10381532; doi:10.3390/life13071484)
Supplement: Supplementary file 1 [file life-13-01484-s001.zip › life-2452305-supplementary.pdf]

Supplementary File S1: Comparison of the momentary moving speed between male and female at representative time points.

Characteristics of subjects	Male (n = 9)	Female (n = 27)	*P* value
MMS (0.01 s)	35.05 ± 37.37	69.41 ± 77.67	0.2420
MMS (0.05 s)	6.85 ± 4.95	9.93 ± 7.09	0.2005
MMS (0.1 s)	3.58 ± 1.92	4.59 ± 2.63	0.4009
MMS (0.5 s)	0.96 ± 0.30	0.89 ± 0.43	0.4650
MMS (1.0 s)	0.60 ± 0.31	0.46 ± 0.25	0.3077
MMS (2.0 s)	0.40 ± 0.31	0.25 ± 0.15	0.2005
α	0.58 ± 0.11	0.46 ± 0.25	0.3077
β	0.91 ± 0.28	1.00 ± 0.30	0.2150

MMS = momentary moving speed. Mann-Whitney U test was used for the statistical analysis.
